# Selection criteria for 3D conformal radiotherapy versus volumetric-modulated arc therapy in high-grade glioma based on normal tissue complication probability of brain

**DOI:** 10.1093/jrr/rry106

**Published:** 2019-01-11

**Authors:** Satoshi Tanabe, Haruna Takahashi, Hirotake Saito, Atsushi Ohta, Toshimichi Nakano, Ryuta Sasamoto, Miki Shioi, Satoru Utsunomiya, Eisuke Abe, Motoki Kaidu, Hidefumi Aoyama

**Affiliations:** 1 Department of Radiation Oncology, Niigata University Medical and Dental Hospital, 1-757 Asahimachi-dori, Chuo-ku, Niigata, Japan; 2 Department of Radiation Technology, Niigata City General Hospital, 463-7 Shumoku, Chuo-ku, Niigata, Japan; 3 Department of Radiology and Radiation Oncology, Niigata University Graduate School of Medical and Dental Sciences, 1-757 Asahimachi-dori, Chuo-ku, Niigata, Japan; 4 Department of Radiological Technology, Niigata University Graduate School of Health Sciences, 2-746 Asahimachi-dori, Chuo-ku, Niigata, Japan

**Keywords:** radiotherapy, high-grade glioma, VMAT, NTCP, selection criteria

## Abstract

There are no quantitative selection criteria for identifying high-grade glioma (HGG) patients who are suited for volumetric-modulated arc therapy (VMAT). This study aimed to develop selection criteria that can be used for the selection of the optimal treatment modality in HGG. We analyzed 20 patients with HGG treated by 3D conformal radiotherapy (3DCRT). First, VMAT plans were created for each patient retrospectively. For each plan, the normal tissue complication probability (*NTCP*) for normal brain was calculated. We then divided the patients based on the *NTCP*s of the 3DCRT plans for normal brain, using the threshold of 5%. We compared the *NTCP*s of the two plans and the gross tumor volumes (*GTV*s) of the two groups. For the *GTV*s, we used receiver operating characteristic curves to identify the cut-off value for predicting *NTCP* < 5%. We determined the respective correlations between the *GTV* and the GTV’s largest cross-sectional diameter and largest cross-sectional area. In the *NTCP* ≥ 5% group, the *NTCP*s for the VMAT plans were significantly lower than those for the 3DCRT plans (*P* = 0.0011). The *NTCP* ≥ 5% group’s *GTV* was significantly larger than that of the *NTCP* < 5% group (*P* = 0.0016), and the cut-off value of the *GTV* was 130.5 cm^3^. The *GTV* was strongly correlated with the *GTV*’s largest cross-sectional diameter (*R*^2^ = 0.82) and largest cross-sectional area (*R*^2^ = 0.94), which produced the cut-off values of 7.5 cm and 41 cm^2^, respectively. It was concluded that VMAT is more appropriate than 3DCRT in cases in which the *GTV* is ≥130.5 cm^3^.

## INTRODUCTION

Post-operative radiotherapy is absolutely essential for high-grade glioma (HGG). With the technical advances in radiotherapy, the use of intensity-modulated radiation therapy (IMRT) and/or volumetric-modulated arc therapy (VMAT) for the radiation of HGGs is increasingly common as a substitute for 3D conformal radiotherapy (3DCRT) [1]. Several studies have reported that IMRT including VMAT can achieve high conformity for the target while reducing the dose to organs at risk (OARs), compared with 3DCRT [2–7]. Wagner *et al.* [2] described their dosimetric comparison of IMRT and 3DCRT plans for 14 consecutive patients with malignant glioma, and they reported that if the planning target volume (*PTV*) is near an OAR, the *PTV* coverage for IMRT is more acceptable than that for 3DCRT. Lorentini *et al.* [3] assessed the clinical dosimetric scenario that could benefit the most from an IMRT plan versus a 3DCRT plan for 17 patients with glioblastoma, and they reported that the higher the number of *PTV*–OARs overlaps, the better the target coverage provided by IMRT compared with a 3DCRT plan. In particular, Sakanaka *et al.* [7] reported that VMAT could reduce the number of monitor units, while maintaining target coverage comparable with that of IMRT. However, it is difficult to use VMAT for all HGG patients, because it requires a longer preparation time and more human resources compared with 3DCRT.

Considering the ease of the preparation for 3DCRT, it has not till now been clear which patients would benefit from undergoing VMAT rather than 3DCRT. To our knowledge, there are no quantitative selection criteria for identifying HGG patients who are suited for VMAT. As HGG can grow rapidly, it is desirable to determine the treatment selection of radiotherapy as early as possible after surgery. In regard to this point, the gross tumor volume (*GTV*) can be identified on diagnostic images at the medical examination. Hence, we conducted the present study to develop quantitative selection criteria, focused on the *GTV*, that could be used for the selection of the optimal treatment modality in HGG, in a comparison of the VMAT plan with the 3DCRT plan.

## METHODS

### Patients

Among 46 consecutive HGG patients who underwent 3DCRT at our institution in the 18-month period from August 2014 to February 2016, those who met the following criteria were included: (i) the patients to whom 3DCRT of 60 Gy in 30 fractions was delivered, and (ii) enhancement of their tumor was observed on the T2-weighted or fluid-attenuated inversion recovery (FLAIR) MRI, and (iii) the tumor was not located in the brainstem. As a result, 20 patients were identified and considered suitable for the further analyses. The clinical characteristics of the 20 patients are summarized in Table 1. There were 12 males and eight females, with a median age of 59 years (range 29–72 years). The median *GTV* was 91.9 cm^3^ (range 14.0–391.5 cm^3^), and the median maximal diameter of the *GTV* was 7.3 cm (range 4.1–11.0 cm). This study was approved by our institutional review board (IRB) (No. 2015–2632). All patients were provided their informed consent under our IRB concerning the use of their data for research purpose.

**Table 1.** Patient characteristics

*n* = 20	
Sex	
Male	12
Female	8
Age	
Median	59
Range	29–72
WHO grade	
Grade III	8
Grade IV	12
Tumor location	
Parietal lobe	2
Temporal lobe	3
Frontal lobe	12
Parietal–temporal	2
Cerebellum	1
*GTV* (cm^3^)	
Median	91.9
Range	14.0–391.5
Largest cross-sectional diameter (cm)	
Median	7.3
Range	4.1–11.0
Largest cross-sectional area (cm^2^)	
Median	38.6
Range	11.7–84.6
Surgery	
Gross total resection	9
Sub-total resection	7
Partial resection	3
Biopsy	1

### Treatment planning simulation in 3DCRT

**Fig. 1.**
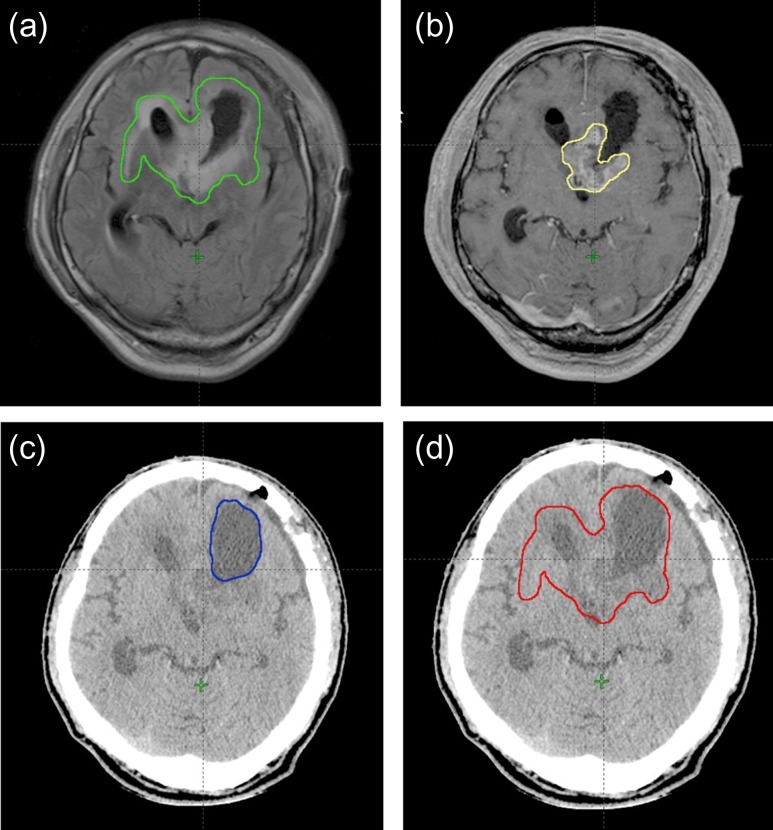
Treatment planning images. (a) The hyper-intense regions on this FLAIR MRI image are outlined in green. (b) The enhanced residual tumor on this MRI image is outlined in yellow. (c) The removal cavity on this CT image is outlined in blue. (d) The *GTV* on this CT image is outlined in red.

A treatment planning simulation in 3DCRT was performed with a 16-slice CT scanner (Lightspeed RT, General Electric, Freiburg, Germany). Radiotherapy (RT) treatment planning was generated with an Eclipse™ ver. 11.0 treatment planning system (Varian Medical Systems, Palo Alto, CA). The dose calculation was performed using the Anisotropic Analytical Algorithm ver. 11.0 in the Eclipse system. The *GTV* included the residual enhancing tumor, the tumor removal cavity, and the hyper-intense regions on the T2-weighted or FLAIR MRI, according to the Radiation Therapy Oncology Group (RTOG) guidelines for target delineation of glioblastoma (Fig. 1) [8].

In the initial plan, a clinical target volume (*CTV*) was created by expanding the *GTV* by 15-mm isotropic margins. The *PTV* was created by expanding the *CTV* by 3-mm isotropic margins. We refer to the *CTV* and the *PTV* in the initial plan as the ‘*CTV*-initial’ and ‘*PTV*-initial’, respectively. In the initial plan, 50 Gy in 25 fractions at the isocenter was delivered with five 6 MV coplanar beams on a Novalis-TX system (Varian Medical Systems and BrainLAB, Feldkirchen, Germany). The gantry angles were set to 0°, 72°, 144°, 218° and 290°. In cases in which the *GTV* was adjacent to critical OARs such as the brainstem and optic nerves, the prescribed dose was reduced to 46 Gy in 23 fractions. The margin between the *PTV*-initial and the ends of the multileaf collimators (MLCs) on the beam’s-eye view was set to 2 mm.

In the boost plan after 50 Gy in 25 fractions, the *CTV* (*CTV*-boost) was created by adding 15-mm isotropic margins around the residual enhancing tumor and the tumor removal cavity. The *PTV* (*PTV*-boost) was created by expanding the *CTV*-boost by 3-mm isotropic margins; 10 Gy in five fractions or 14 Gy in seven fractions at the isocenter were delivered with two 6 MV opposing beams in order to spare the OARs for each patient. The sum of the radiation doses delivered in the initial and the boost plans was 60 Gy in 30 fractions in total.

### Treatment planning simulation in VMAT

For all HGG patients who satisfied the inclusion criteria, we retrospectively generated one-arc 6 MV VMAT plans (gantry angle: 181° to 179°) by using the simultaneous integrated boost method. A collimator angle was set to 30° in order to minimize the tongue-and-groove effect. The prescribed doses were 60 Gy in 30 fractions for the *PTV*-boost and 50 Gy in 30 fractions for the *PTV*-initial. All plans were designed so that 50% of the *PTV*-boost received the prescription dose, at least 99% of each *PTV* received 90% of the prescription dose, and no more than 2% of the *PTV* received 107% of the prescription dose. All OARs were evaluated as the planning OAR volume (*PRV*) in this study. For all *PRV*s, the following constraints were used: optic chiasm *D*_2%_ (dose delivered to 2% of the considered structure volume) < 50 Gy, eyes *D*_2%_ < 45 Gy, optic nerves *D*_2%_ < 50 Gy, brainstem *D*_2%_ < 54 Gy, lens mean dose < 6 Gy, and internal ears mean dose < 45 Gy. All plans were approved prior to the conformity of the *PTV* and normal brain.

### Evaluation of the normal tissue complication probability of normal brain

In this study, the normal brain was defined as the whole brain minus the *GTV*. We evaluated the normal tissue complication probability (*NTCP*) of normal brain by using the Lyman–Kutcher–Burman (LKB) model in Eqs. (1) and (2):

(1) $$\mathrm{NTCP}=\frac{1}{\sqrt{2\pi }}\int_{-\infty }^{t}e^{-\frac{x^{2}}{2}}\mathrm{dx}$$

(2) $$t=\frac{D-{\mathrm{TD}}_{50}}{m\times {\mathrm{TD}}_{50}},$$

where *TD*_50_ is the tolerance dose for a 50% complication probability for uniform doses to the organ, and *m* is a dimensionless parameter for determining the slope of the complication probability according to the dose curve [9].

For the uniform dose *D* in Eq. (2), we used the generalized equivalent uniform dose (*gEUD*), as shown in Eq. (3):

(3) $$\mathrm{gEUD}=(\sum_{i=1}^{N}v_{i}D_{i}^{a}){}^{\frac{1}{a}}$$

where *D*i is the dose for each bin in a differential dose–volume histogram (DVH), *v*_i_ is the volume in a specific dose bin i, and *N* is the unequal fractional sub-volume. The ‘*a*’ value is a parameter equal to 1/*n*, in which *n* represents the volume dependence of the complication probability [7]. We adopted the following parameters to evaluate the radiation-induced brain necrosis as an end point: *TD*_50_ = 60, *m* = 0.15, *a* = 4 [10].

### Evaluation

**Fig. 2.**
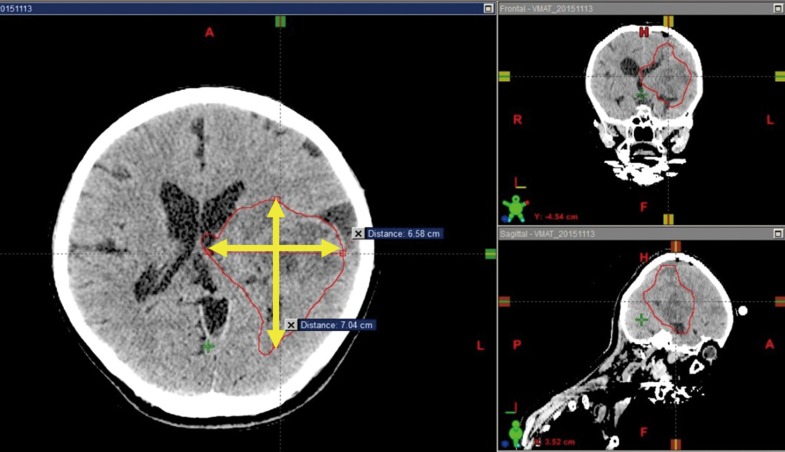
The largest cross-sectional diameter of the *GTV* and the largest diameter perpendicular to it. The *GTV* on this CT image is outlined in red.

The percentage volume of the normal brain receiving at least 5–60 Gy (V_5 Gy_–V_60 Gy_), *D*_2%_, mean dose, *gEUD* and *NTCP* were obtained from DVHs for each patient in order to compare the 3DCRT and VMAT plans (60 Gy in 30 fractions). In addition to the normal brain, the DVHs of the *PTV*-boost and the *PRV*s for the brainstem, optic chiasm, optic nerve, eye and lens were analyzed. The *PTV*-boost was assessed by calculating the *D*_95%_, *D*_98%_, *D*_2%_, *V*_90%_, *V*_95%_, homogeneity index defined as (*D*_2%_–*D*_98%_)/*D*_50%_, and conformity index (*CI*). For the determination of the *CI*, we used the following formula:

(4) $$\mathrm{Conformity}\;\mathrm{index}=\frac{{\mathrm{TV}}_{\mathrm{RI}}}{\mathrm{TV}}\times \frac{{\mathrm{TV}}_{\mathrm{RI}}}{V_{\mathrm{RI}}}$$

where *TV* is the target volume, *V*_RI_ is the volume of the reference isodose, and *TV*_RI_ is the target volume covered by the reference isodose [11]. In the present study, we defined the reference isodose as the 95% isodose. In clinical practice, the *TD*5/5 (the tolerance dose for a 5% risk of developing ≥Grade 3 toxicities within 5 years) is frequently used, and we therefore consider the *TD*5/5 appropriate for setting the cut-off value of the *NTCP* as 5% [12]. We divided the patients into two groups based on the *NTCP* of normal brain in each 3DCRT plan by using the threshold of 5% (*NTCP* < 5% and *NTCP*≥5% groups), and we then compared the *NTCP*s for the 3DCRT and VMAT plans between these two groups. We also compared the *GTV* between the groups. The correlations between the *GTV* and the largest cross-sectional diameter of the *GTV* and between the *GTV* and the largest cross-sectional area (largest cross-sectional diameter by the largest diameter perpendicular to it) of the *GTV* were evaluated in reference to Wen *et al.* (Fig. 2) [13].

### Statistical analysis

The Wilcoxon signed rank test was used for comparisons of 3DCRT and VMAT, and of the *GTV* for the *NTCP* < 5% and *NTCP* ≥ 5% groups. In order to evaluate the prediction accuracy of the *GTV* for *NTCP* < 5%, the area under the curve (AUC) of a receiver operating characteristic (ROC) curve was used. The best cut-off value for predicting *NTCP* < 5% of ROC curves was determined by the Youden’s index, defined as the point that (sensitivity + specificity – 1) becomes the maximum [14]. A *P*-value of <0.05 was considered statistically significant. All statistical analyses were performed using the JMP ver. 11 program (SAS, Cary, NC), R statistical software version 3.0.2 (the R Foundation for Statistical Computing, Vienna, Austria), and EZR (a graphical user interface for R, Saitama Medical Center, Jichi Medical University, Saitama, Japan).

## RESULTS

### Dosimetric comparison between the 3DCRT and VMAT plans

Table 2 shows the results of the dosimetric comparison between the 3DCRT and VMAT plans. With respect to the *PTV*-boost, the median *CI* value in the VMAT plan (0.93) was significantly superior to that in the 3DCRT plan (0.59) (*P* < 0.001), although there were no significant differences between the 3DCRT and VMAT plans in the median values of *D*_95%_ (57.3 Gy vs 57.2 Gy, *P* = 0.85), *D*_98%_ (54.7 Gy vs 56.3 Gy, *P* = 0.23), *D*_2%_ (62.5 Gy vs 63.0 Gy, *P* = 0.14), *V*_90%_ (98.2% vs 99.7%, *P* = 0.079), *V*_95%_ (95.9% vs 95.7%, *P* = 0.90) and *HI* (0.14 vs 0.11, *P* = 0.33), respectively.

**Table 2.** Comparison of dosimetric parameters for 3DCRT and VMAT plans

Structure	3D-CRT median (range)	VMAT median (range)	*P*-value
*PTV*-boost (60 Gy)			
D_95%_ (Gy)	57.3 (46.7–60.0)	57.2 (50.6–58.0)	0.85
D_98%_ (Gy)	54.7 (45.4–59.8)	56.3 (49.4–57.5)	0.23
D_2%_ (Gy)	62.5 (60.7–64.5)	63.0 (62.0–63.8)	0.14
*V*_90%_ (%)	98.2 (73.7–100)	99.7 (82.7–100)	0.079
*V*_95%_ (%)	95.9 (69.6–100)	95.7 (76.7–99.3)	0.90
HI	0.14 (0.026–0.29)	0.11 (0.085–0.24)	0.33
*CI*	0.59 (0.43–0.77)	0.93 (0.69–0.96)	<0.001*
Normal brain			
*V*_5 Gy_ (%)	94.1 (68.7–100)	94.5 (74.5–100)	0.97
*V*_10 Gy_ (%)	88.2 (59.0–99.8)	86.9 (64.3–99.8)	0.88
*V*_15 Gy_ (%)	82.0 (56.1–97.8)	74.6 (49.1–95.2)	0.48
*V*_20 Gy_ (%)	74.6 (45.9–96.0)	63.7 (36.2–87.1)	0.083
*V*_25 Gy_ (%)	63.0 (27.0–90.5)	51.2 (26.2–78.3)	0.33
*V*_30 Gy_ (%)	49.7 (20.4–77.5)	41.1 (19.2–67.6)	0.28
*V*_35 Gy_ (%)	43.4 (16.7–67.4)	33.5 (14.7–55.9)	0.19
*V*_40 Gy_ (%)	37.5 (13.5–58.1)	27.8 (11.9–46.1)	0.14
*V*_45 Gy_ (%)	32.6 (11.3–50.5)	23.7 (9.88–39.5)	0.15
*V*_50 Gy_ (%)	24.2 (6.74–43.6)	16.7 (7.58–31.9)	0.14
*V*_55 Gy_ (%)	18.9 (5.20–37.1)	10.9 (3.85–20.9)	0.0032*
*V*_60 Gy_ (%)	10.3 (1.87–24.9)	4.94 (1.14–7.47)	<0.001*
D_2%_ (%)	62.1 (59.9–63.9)	61.3 (59.2–62.1)	0.0068*
Mean dose (Gy)	31.6 (20.2–43.9)	27.7 (19.1–38.8)	0.26
*gEUD*	43.3 (34.3–49.9)	39.3 (33.3–45.9)	0.06
*NTCP* (%)	3.2 (0.21–13.2)	1.1 (0.15–5.81)	0.06
<5	1.2 (0.21–4.67)	0.8 (0.15–3.02)	0.09
≥5	9.6 (5.40–13.2)	4.0 (2.80–5.81)	0.0011*
Brainstem_*PRV*			
D_2%_ (Gy)	47.5 (30.4–57.4)	49.8 (27.0–53.8)	0.67
Optic chiasm_*PRV*			
D_2%_ (Gy)	46.6 (5.16–54.5)	48.5 (6.07–49.9)	0.17
Optic nerve_PRV			
D_2%_ (Gy)	45.8 (2.86–54.4)	46.2 (2.77–49.8)	0.17
Eye_PRV			
D_2%_ (Gy)	21.2 (1.29–46.6)	21.3 (1.29–40.3)	0.86
Lens_PRV			
Mean dose (Gy)	2.6 (0.70–6.08)	3.2 (0.0–5.64)	0.43

D_*x*%_ = the radiation dose delivered to *x*% of the structure, *V*_*x*%_ = the percentage of the volume of the structure that receives ≥*x*% of the prescribed dose, HI = homogeneity index, *CI* = conformity index, *gEUD* = generalized equivalent uniform dose, *NTCP* = normal tissue complication probability.

There were no significant differences between the 3DCRT and VMAT plans in the median value of the *gEUD* (43.3 Gy vs 39.3 Gy, *P* = 0.06) or the *NTCP*s for normal brain (3.2% vs 1.1%, *P* = 0.06), respectively, on the whole. In contrast, in the *NTCP* ≥ 5% group, the *NTCP*s for the VMAT plans (4.0%) were significantly lower than the *NTCP*s for the 3DCRT plans (9.6%) (*P* = 0.001). The VMAT plan had clear advantages with respect to the differences between the *V*_55 Gy_, *V*_60 Gy_ and *D*_2%_ for normal brain. There were no significant differences in the median value of the mean dose and *V*_5 Gy_–*V*_50 Gy_ for normal brain and *D*_2%_ for each *PRV* in the 3DCRT or VMAT plans.

### Determination of the cut-off value of the *GTV*

**Fig. 3.**
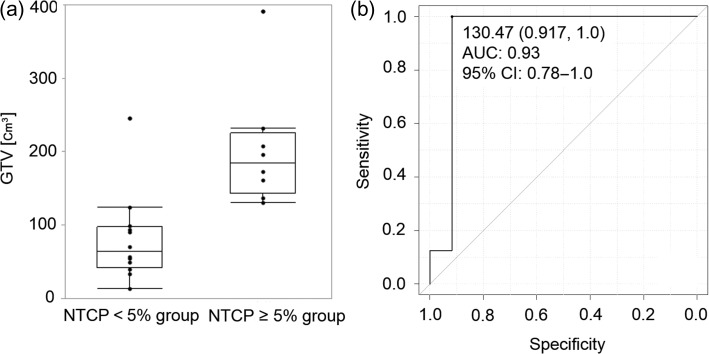
The distribution of the *GTV* values in the *NTCP* < 5% group and *NTCP* ≥ 5% group (a), and the ROC curve used to identify the *GTV* cut-off value (b).

The distribution of the *GTV* values between the *NTCP* < 5% group and the *NTCP* ≥ 5% group is illustrated in Fig. 3a. The median value of the *GTV* in the *NTCP*≥5% group (149.5 cm^3^, range 45.4–391.5 cm^3^) was significantly larger than that in the *NTCP* < 5% group (64.2 cm^3^, range 14.0–246.4 cm^3^) (*P* = 0.0016). As shown in Fig. 3b, the cut-off value of the *GTV* was 130.5 cm^3^ (AUC 0.93, 95% *CI* 0.78–1.0).

**Fig. 4.**
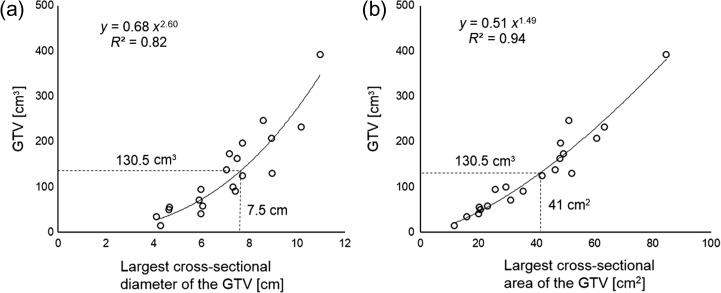
The correlation between the *GTV* and the largest cross-sectional diameter of the *GTV* (a), and the largest cross-sectional area of the *GTV* (b).

Figure 4 shows the relationship between the *GTV* and the largest cross-sectional diameter of the *GTV* (Fig. 4a) and the largest cross-sectional area of the *GTV* (Fig. 4b). The *GTV* was strongly correlated with both the largest cross-sectional diameter of the *GTV* (*R*^2^ = 0.82) and the largest cross-sectional area (*R*^2^ = 0.94), which produced the cut-off values of 7.5 cm and 41 cm^2^.

## DISCUSSION

Our analyses revealed two important findings. First, the high *NTCP* of normal tissue is associated with a large *GTV*. Second, compared with 3DCRT, the use of VMAT can significantly reduce the *NTCP* of normal brain for plans with a high *NTCP*. These findings can form the basis of concise and objective selection criteria for radiotherapy techniques in HGG patients.

The *NTCP* is calculated to estimate the risk of normal tissue complications based on the dose distribution. It is expressed as an approximation formula of the cumulative normal distribution of dose and volume histograms. The integrated parameters vary depending on each organ and complication. In the LKB model, the dose distribution is transformed into the generalized equivalent uniform dose (*gEUD*), and the *gEUD* is then used to calculate the *NTCP*.

Our present findings demonstrated that the *NTCP* value for normal brain can be significantly decreased by the use of VMAT in the *NTCP* ≥ 5% group, although no significant difference was found in the *NTCP* < 5% group. Thus, in terms of radiation-induced brain necrosis, VMAT has few advantages over 3DCRT in patients with an *NTCP* value < 5%. Considering the preparatory period for radiotherapy and the human resources required, the selection of 3DCRT can be justified.

In clinical practice, the choice of the radiotherapy technique is usually made as part of the outpatient service. However, it is impossible to evaluate the *NTCP* for normal brain at that stage. In this study, we observed the patient’s *GTV* values and identified the *GTV* that produced the cut-off value of 5% *NTCP*, based on our hypothesis that this could be a decisive criterion for the selection between 3DCRT and VMAT. As shown in Fig. 4, the *GTV* was strongly correlated with the largest cross-sectional diameter of the *GTV* (*R*^2^ = 0.82) and the largest cross-sectional area (*R*^2^ = 0.94). Our proposed cut-off value is useful in that the choice between 3DCRT and VMAT can thus be made before CT images are acquired for treatment planning, because these parameters are easily assessed in the diagnostic MRI images.

Several studies have reported that intensity-modulated radiotherapy (IMRT) including VMAT is superior to 3DCRT in radiotherapy for HGG [2–7]. As mentioned in the Introduction section, Lorentini *et al.* [2] reported that the higher the number of *PTV*–OARs overlaps, the better the target coverage provided by IMRT compared with a 3DCRT plan. However, their study did not take the volume of the *PTV*–OAR overlap into account. Moreover, a treatment planning system was needed in order to measure the volume of the overlap in that study. Thus, the use of these methods might be difficult in the first medical examination, because there is no quantitative information about treatment planning. In contrast, in the present study, we assessed the selection criteria for VMAT using the *NTCP* for normal brain as a quantitative index prior to the treatment planning process.

In a similar study by MacDonald *et al.* [4], IMRT significantly lowered the *NTCP* for normal brain from 0.23% with 3DCRT to 0.043% (*P* = 0.003). The lower *NTCP* values in the MacDonald study compared with those in the present study are due to the difference in the definition of normal brain (defined as brain minus *PTV* in the MacDonald study). The study by Hermanto *et al.* [5] evaluated the normal brain defined as two different volumes: brain minus *PTV* and brain minus *GTV*, assessing the integral dose to normal brain for 20 HGG patients. Although more research comparing the actual occurrence rate for brain necrosis and *NTCP* is necessary in order to precisely evaluate the risk of radiation-induced brain necrosis, it is reasonable to propose that ‘brain minus *GTV*’ is preferable to ‘brain minus *PTV*’ because the set-up margin differs between institutions. We eventually identified ‘the largest cross-sectional diameter of the *GTV*’ and/or ‘the largest cross-sectional area of the *GTV*’ on the diagnostic images as the selection criteria for VMAT. Therefore, our proposed criteria are clinically relevant because each value can be easily obtained by measuring it on the diagnostic imaging at an outpatient service.

There have been no randomized controlled trials comparing the clinical outcomes of IMRT with those of 3DCRT in HGG. In prostate or head and neck cancers, the results of dosimetric studies correlate with the incidence of actual adverse events [15, 16]. In HGG patients, it is feasible to consider the application of IMRT based on the findings from radiotherapy simulation studies. Our present findings may useful for building the theoretical basis for future prospective clinical trials.

Our study has limitations that are inherent in simulation studies. First, we adopted only the LKB model in order to evaluate the *NTCP*, which is largely influenced by the extrapolated parameters. However, the parameters we adopted for the LKB model are also used in many studies, and this type of vulnerability might be encountered in other studies relevant to *NTCP*. Second, we did not investigate the influence of the *GTV* location. In cases in which the *GTV* is smaller than that in the criteria proposed herein, and in cases in which the *GTV* is adjacent to critical OARs, the use of VMAT may be more effective compared with 3DCRT. Third, we did not consider the fraction schemes, and no correction for fractionations were applied in order to compare the *NTCP* between 3DCRT and VMAT plans. Therefore, our proposed cut-off value should be regarded as only part of the basis for decision-making in clinical settings.

## CONCLUSION

We developed quantitative selection criteria for VMAT in HGG in terms of radiation-induced brain necrosis. Our findings show that VMAT is more appropriate than 3DCRT in cases in which the *GTV* is ≥130.5 cm^3^, which corresponds to the threshold of 7.5 cm in the largest cross-sectional diameter of the *GTV* and to 41 cm^2^ in the largest cross-sectional area of the *GTV* on diagnostic images.
